# RV‐LV volumetric relationship during graded supine exercise in healthy adults assessed by cardiovascular magnetic resonance

**DOI:** 10.14814/phy2.71206

**Published:** 2026-11-06

**Authors:** Megumi Ikeda, Michinari Hieda, Keimei Yoshida, Misaki Imabayashi, Daisuke Toyomura, Ryo Izumi, Akiko Nishizaki, Tatsuhiro Wada, Yuya Saito, Chiaki Tokunaga, Kei Nishikawa, Saki Kurokawa, Yuya Fukudome, Takuya Hino, Yuzo Yamasaki, Kazuya Hosokawa, Mitsuhiro Fukata, Kousei Ishigami, Kohtaro Abe, Koichi Akashi

**Affiliations:** ^1^ Medicine and Biosystemic Science, Graduate School of Medical Sciences Kyushu University Fukuoka Japan; ^2^ The Second Department of Internal Medicine, Department of Cardiology and Nephrology University of Occupational and Environmental Health, Japan Fukuoka Japan; ^3^ Cardiovascular Medicine, Graduate School of Medical Sciences Kyushu University Fukuoka Japan; ^4^ Pediatrics, Graduate School of Medical Sciences Kyushu University Fukuoka Japan; ^5^ Division of Radiology, Department of Medical Technology Kyushu University Hospital Fukuoka Japan; ^6^ Clinical Radiology, Graduate School of Medical Sciences Kyushu University Fukuoka Japan

**Keywords:** exercise cardiovascular magnetic resonance, right‐to‐left ventricular volume ratio, RV‐LV volumetric relationship

## Abstract

Exercise cardiovascular magnetic resonance imaging (exCMR) enables quantitative assessment of biventricular responses during physiological stress. We aimed to characterize right ventricular (RV)‐left ventricular (LV) volumetric relationships in healthy adults using exCMR. Eighteen healthy adults (9 males, 9 females) underwent exCMR. Electrocardiography‐gated short‐axis cine and phase‐contrast flow images were obtained at rest, at a target heart rate of 100 beats/min (Ex1), and at 40% of peak work rate determined by cardiopulmonary exercise testing (Ex2). The RV‐LV volumetric relationship was operationally represented by the RV/LV end‐diastolic and end‐systolic volume ratios derived from simultaneous cine volumetry. All participants completed the protocol, and all cine and phase‐contrast datasets were analyzed. During exercise, stroke volume increased in both ventricles predominantly through a reduction in end‐systolic volume (ESV), whereas end‐diastolic volume (EDV) changed little. Cine‐derived LV and RV stroke volumes showed internal concordance with phase‐contrast flow‐derived measurements. The RV/LV EDV ratio (1.03, 1.03, and 1.03 at rest, Ex1, and Ex2, respectively) and RV/LV ESV ratio (1.09, 1.09, and 1.08 at rest, Ex1, and Ex2, respectively) remained stable across stages. Healthy adults maintained a stable relative RV‐LV volumetric relationship during low‐to‐moderate intensity supine exercise. These findings provide quantitative physiological reference data for future disease‐focused studies.

## Graphical abstract

We assessed biventricular volumes in healthy adults using exercise CMR. RV/LV EDV and ESV ratios remained stable during low‐to‐moderate intensity supine exercise.

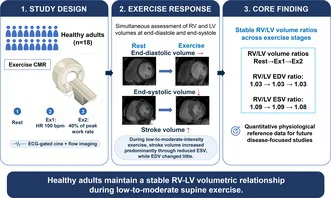

## 1 INTRODUCTION

The left ventricle (LV) and right ventricle (RV) are anatomically and functionally connected, and a coordinated response from both ventricles is required to maintain adequate circulation. Interventricular interactions refer to the mechanical and functional interdependence between the RV and LV arising from myocardial continuity, the shared interventricular septum, and pericardial constraint (Chung et al., 1997; Olsen et al., 1983). Although LV and RV volumes are influenced by multiple physiological factors, they remain closely linked to systemic and pulmonary vascular resistance, respectively, particularly during systole. Consequently, coordinated changes in RV and LV chamber volumes may reflect integrated cardiovascular adaptation during exercise. Cardiovascular magnetic resonance imaging (CMR) provides accurate and highly reproducible quantification of biventricular volumes and function (Ponikowski et al., 2016; Schulz‐Menger et al., 2020).

The marked increase in oxygen demand of the skeletal muscles during exercise requires augmentation of cardiac output. To increase stroke volume while maintaining forward flow through the series circulation, coordinated adaptation of the pulmonary and systemic circulation is required. Increased LV filling pressure during exercise stress may result in secondary pulmonary hypertension, further exacerbating adverse ventricular interactions (Schwarz et al., 2013). In contrast, elevated RV pressure, which models acute pulmonary embolism or pulmonary hypertension, impairs ventricular interaction with the LV via interventricular shift. This adverse interaction reduces LV preload and stroke volume (Belenkie et al., 1988). Therefore, simultaneous assessment of both ventricular volumes during exercise may provide a quantitative perspective on coordinated biventricular adaptation during physiological stress.

Echocardiography remains the most used imaging modality to evaluate cardiovascular function and exercise performance during stress (Gibbons et al., 1997; Lancellotti et al., 2016). However, accurate simultaneous assessment of RV and LV volumes by echocardiography is challenging, particularly for the geometrically complex RV, owing to limitations in endocardial delineation and volumetric quantification (Lang et al., 2015). Exercise CMR (exCMR) enables biventricular volumetric assessment during physiological stress (Craven et al., 2020). Previous exCMR studies have characterized biventricular volumetric responses across graded exercise intensities (Claessen et al., 2014; la Gerche et al., 2013). In light of these prior studies, the present study focused on the relative RV‐LV volumetric relationship during standardized submaximal supine exercise using ECG‐gated exCMR.

Therefore, this study aimed to quantify biventricular volumetric adaptation using exCMR in healthy adults. We operationally represented RV‐LV volumetric relationships by RV/LV end‐diastolic volume (EDV) and RV/LV end‐systolic volume (ESV) ratios. We hypothesized that healthy adults would maintain a stable RV‐LV volumetric relationship during exercise. By characterizing physiological responses in healthy adults, this study aimed to provide a physiological foundation for future disease‐focused investigations of altered biventricular adaptation.

## 2 MATERIALS AND METHODS

### 2.1 Study design and population

This single‐center, prospective, observational study was approved by the Institutional Ethics Committee of Kyushu University (No. 23427) and conformed to the Declaration of Helsinki. Written informed consent was obtained from each participant. Informed consent for publication of the anonymized technical images shown in Figure 1 and the graphical abstract was obtained from the participant. The institutional authors controlled all data, and the funders had no role in the study design.

**FIGURE 1:**
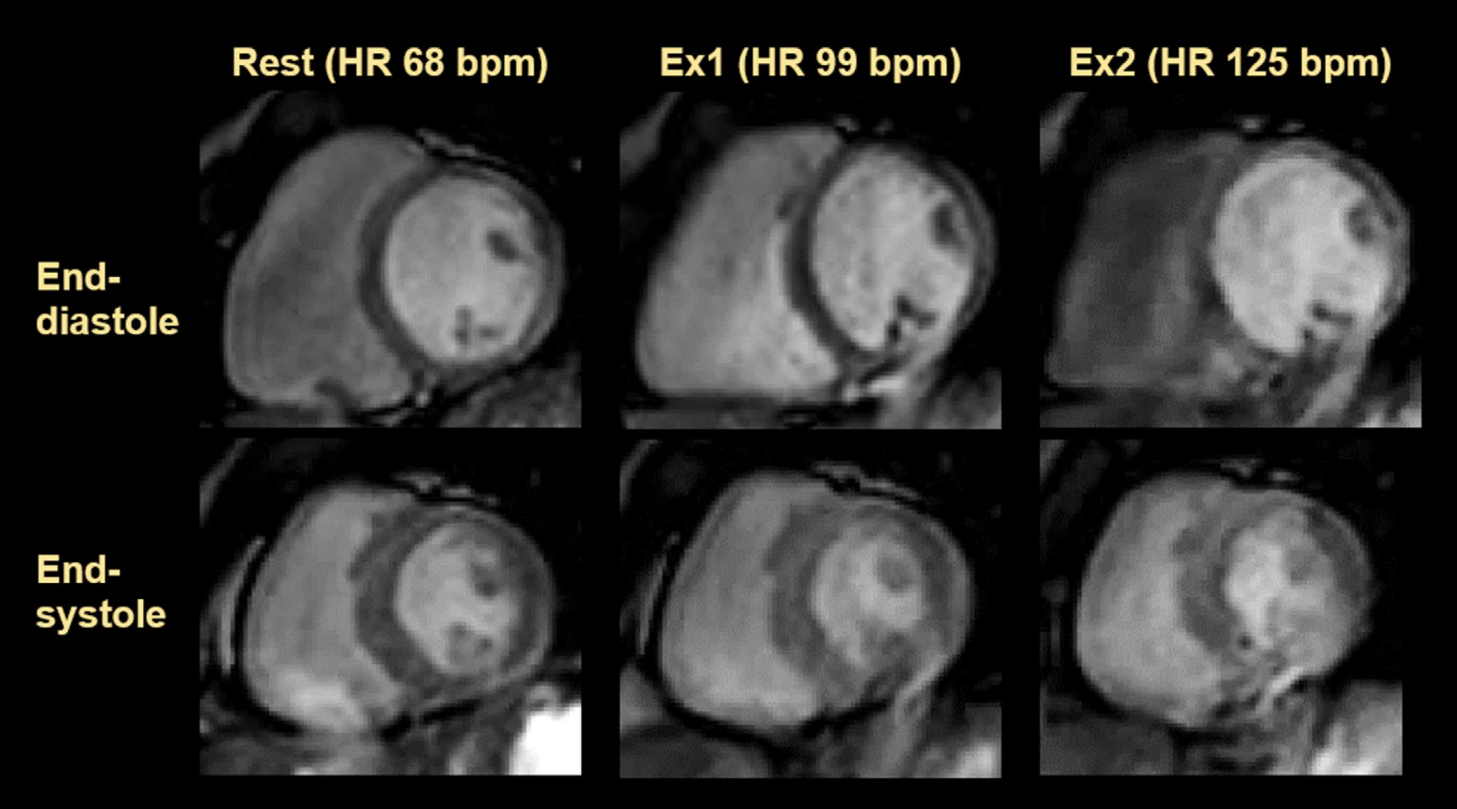
Representative short‐axis cine images in the end‐diastolic and end‐systolic phases at rest and during exercise. Cine short‐axis images were obtained at the mid‐ventricle. The endocardial border is clearly identifiable even during exercise. Informed consent for publication of these anonymized images was obtained from the participant. Ex1, exercise at a target heart rate of 100 beats/min; Ex2, exercise at 40% of peak work rate; HR, heart rate.

Healthy individuals without cardiopulmonary diseases who volunteered to participate after responding to local advertisements were evaluated. A total of 19 healthy volunteers (10 males and 9 females) were recruited between August 2023 and July 2024. One participant was excluded after an initial screening echocardiography revealed a patent foramen ovale. Ultimately, 18 healthy volunteers were included in the analysis. Standard transthoracic echocardiography was performed at screening by an experienced sonographer and reviewed by a cardiologist to confirm normal cardiac structure and function before enrollment. Screening assessed chamber size and systolic function, significant valvular disease, Doppler findings suggestive of pulmonary hypertension, and intracardiac shunt. Participants with abnormal findings were excluded. Exercise tolerance was assessed using a cardiopulmonary exercise test on an upright cycle ergometer performed 2 weeks before CMR.

### 2.2 Exercise protocol

The exercise workload was applied using a supine ergometer stress test performed with an MRI‐compatible cycle ergometer (Lode‐BV, Netherlands). Images were acquired at three timepoints: at rest, during loading targeting a heart rate (HR) of 100 beats/min (exercise 1 [Ex1]), and at 40% of the peak work rate achieved during cardiopulmonary exercise testing (CPX) (exercise 2 [Ex2]). The HR‐targeted stage was selected to provide a standardized heart‐rate target across participants, whereas 40% of peak work rate represented an individualized relative exercise intensity. These complementary stages were used to characterize biventricular volumetric responses under two distinct submaximal physiological conditions. Exercise stress echocardiography and right heart catheterization have been used based on previous reports of their suitability for examining cardiac contractile and diastolic properties (Sarma et al., 2023). An HR of 100 was selected in those studies to standardize submaximal physiological stress and enable comparison of systolic and diastolic responses between groups. Based on previous exercise‐stress studies using a target HR of 100 beats/min as a standardized submaximal exercise condition, this target was adopted for Ex1 in the present study (Sarma et al., 2023). We selected 40% of the peak work rate from upright CPX because supine exercise corresponds to approximately 66%–83% of the upright load (Le et al., 2017; Wehrle et al., 2021), making this intensity moderate and safe.

Rest images were obtained in the supine position before the participants placed their feet on the pedals to minimize preload changes related to leg positioning and the skeletal‐muscle pump. The participants placed their feet on the ergometer pedals immediately before the exercise load. They first pedaled for approximately 1 min at a 0‐watt load to familiarize themselves with exercising in a supine position, followed by image acquisition at two incremental ramp loads. After adjusting the ergometer to each load, the exercise was continued for at least 2 min, and image acquisition was initiated when the HR was stable. Each stage of image acquisition lasted approximately 3 min.

### 2.3 CMR protocol

CMR images were acquired using a 3.0 T MRI (Ingenia 3.0 T or Ingenia Elition X 3.0 T; Philips Healthcare, Best, Netherlands). A short‐axis cine was used to assess the LV and RV volumes. Cine imaging was performed using a single‐shot balanced fast field echo. Phase‐contrast (PC) imaging using turbo‐field echo planar imaging was performed to determine aortic and pulmonary blood flow. Imaging planes were prescribed perpendicular to the vessel course. Aortic blood flow was evaluated using PC images of the ascending aorta beyond the origin of the coronary arteries. Meanwhile, pulmonary artery blood flow was assessed using PC images of the main pulmonary artery. Cine and PC images were obtained using electrocardiographic (ECG) gating and free‐breathing. When the correct ECG signals could not be obtained because of body movement or elevated heart rate (HR), imaging was performed with peripheral pulse gating.

Short‐axis cine imaging was performed under the following parameters: field of view (FOV): 380 × 380 mm, acquisition matrix = 152 × 152, slice thickness = 10 mm, reconstructed voxel size: 1.1 × 1.1 × 10 mm, flip angle (FA) = 30°, sensitivity encoding factor: 4, repetition time (TR): 2.1 msec, echo time (TE): 1.0 msec, and acquisition time: 1 slice per 1 heartbeat (2 heartbeats required: 1 heartbeat for data acquisition and 1 heartbeat for preparation time for subsequent slice acquisition; a 12‐slice acquisition required 24 s at an HR of 60 beats per minute [bpm]). Meanwhile, PC imaging was performed using the following parameters: FOV = 350 × 350 mm, acquisition matrix: 128 × 128, slice thickness = 10 mm, reconstructed voxel size: 1.6 × 1.6 × 10 mm, FA: 20, turbo field echo factor: 3, echo‐planar imaging factor: 3, TR: 7.1 msec, TE: 3.7 msec, velocity encoding: 250 cm/s, number of slices: 1, and acquisition time: 3 heartbeats.

### 2.4 Image analysis

Image analysis was performed by a cardiologist (M.I.) using the Aquarius iNtuition 4.4.14. P1.283 software (TeraRecon, Durham, NC). Both the LV and RV end‐diastole and end‐systole were determined by visual assessment. LV volumes were calculated excluding the papillary muscles, and LV mass was calculated including them. LV and RV contour tracing was independently reviewed and confirmed by a second physician (K.Y.). For phase‐contrast flow analysis, vessel contours were automatically generated throughout the cardiac cycle and manually corrected when necessary. Stationary tissue background correction was performed before flow quantification.

LV EDV, LV ESV, RV EDV, RV ESV, stroke volume (SV), ejection fraction, aortic flow‐derived SV, and pulmonary flow‐derived SV were quantified at rest and during each exercise stage. In this study, we operationally represented the relative RV‐LV volumetric relationship as RV/LV EDV and RV/LV ESV ratios derived from simultaneously acquired biventricular cine images. These ratios were intended to characterize relative biventricular volumetric adaptation during exercise rather than directly quantify septal or pericardial mechanics. In addition, aortic and pulmonary artery phase‐contrast stroke volumes were analyzed to assess internal concordance of volumetric stroke volume estimates.

### 2.5 Statistical analysis

All values are reported as the mean ± standard deviation. Repeated‐measures one‐way analysis of variance with Tukey's post hoc test was used to compare and evaluate imaging analysis values at rest and during the exercise stages. For pairwise changes in RV/LV EDV and ESV ratios, mean paired differences with 95% confidence intervals and standardized effect sizes for paired data (Cohen's *d*_z_) were calculated. All statistical analyses were performed using Prism 10.1.2 and JMP Pro 17.0.0. *p* < 0.05 was considered significant.

## 3 RESULTS

Screening echocardiography demonstrated normal cardiac structure and function, and CPX indicated normal exercise tolerance in all participants. Baseline characteristics of the 18 healthy volunteers are presented in Table 1. Sex‐stratified participant characteristics and stage‐specific biventricular volumetric measurements are provided in Tables S1 and S2, respectively. All the participants completed the protocol without complications. All cine and PC datasets were of sufficient quality for quantitative analysis. Cine images were reconstructed into 20 cardiac phases at rest, with a median of 20 (range, 17–20) phases at Ex1 and 18 (range, 15–20) phases at Ex2. All participants were imaged using ECG gating at rest and Ex1; peripheral pulse gating was required in 3 of 18 participants (17%) at Ex2. Representative images are shown in Figure 1.

**TABLE 1:** Baseline characteristics and echocardiography/cardiopulmonary exercise test findings.

Baseline characteristics	*n* = 18
Age	36 ± 11
Male/female	9/9
Height (cm)	165.0 ± 8.2
Weight (kg)	59.7 ± 10.8
BSA (m^2^)	1.65 ± 0.17
CPX	
RER	1.29 ± 0.08
Peak HR (beat/min)	178 ± 12
Peak work rate (W)	162 ± 48
AT (W)	85 ± 20
Peak VO_2_ (mL/kg/min)	30.4 ± 6.6
Pred peak VO_2_ (%)	99.7 ± 17.3
VE/VCO_2_ slope	26.8 ± 3.4
ΔVO_2_/ΔWR (mL/min/W)	9.5 ± 1.2
Echocardiography	
LVDd (mm)	46 ± 4
LVDs (mm)	29 ± 3
LVEF (MOD) (%)	62 ± 7
LAVi (mL/m^2^)	27 ± 4.5
E/A	1.7 ± 0.6
E/e'	7.6 ± 2.9
TAPSE (mm)	24 ± 3
FAC (%)	42.7 ± 6.1

*Note*: Values are presented as the mean ± SD.

Abbreviations: AT, anaerobic threshold; BSA, body surface area; CPX, cardiopulmonary exercise testing; E/A, ratio of early diastolic to atrial filling velocity of transmitral flow; E/e', ratio of early mitral inflow velocity to early diastolic mitral annular velocity; FAC, fractional area change; LAVi, left atrial volume index; LVDd, left ventricular end‐diastolic diameter; LVDs, left ventricular end‐systolic diameter; LVEF (MOD), left ventricular ejection fraction (Modified Simpson method); Peak HR, peak heart rate; Peak VO_2_, peak oxygen uptake; Pred peak VO_2_, predicted peak oxygen uptake; RER, respiratory exchange ratio; TAPSE, tricuspid annular plane systolic excursion; VE/VCO_2_ slope, ventilatory equivalent for carbon dioxide slope; ΔVO_2_/ΔWR, change in oxygen uptake per change in work rate.

LV and RV volumetric responses at rest and during exercise are summarized in Figure 2, whereas detailed ventricular volumetric and PC flow measurements are provided in Table 2. In both ventricles, stroke volume increased during exercise, predominantly through a reduction in ESV, whereas EDV changed little over the exercise range studied. Volumetric stroke volumes were concordant with stroke volume estimates derived from PC imaging of the ascending aorta and main pulmonary artery.

**FIGURE 2:**
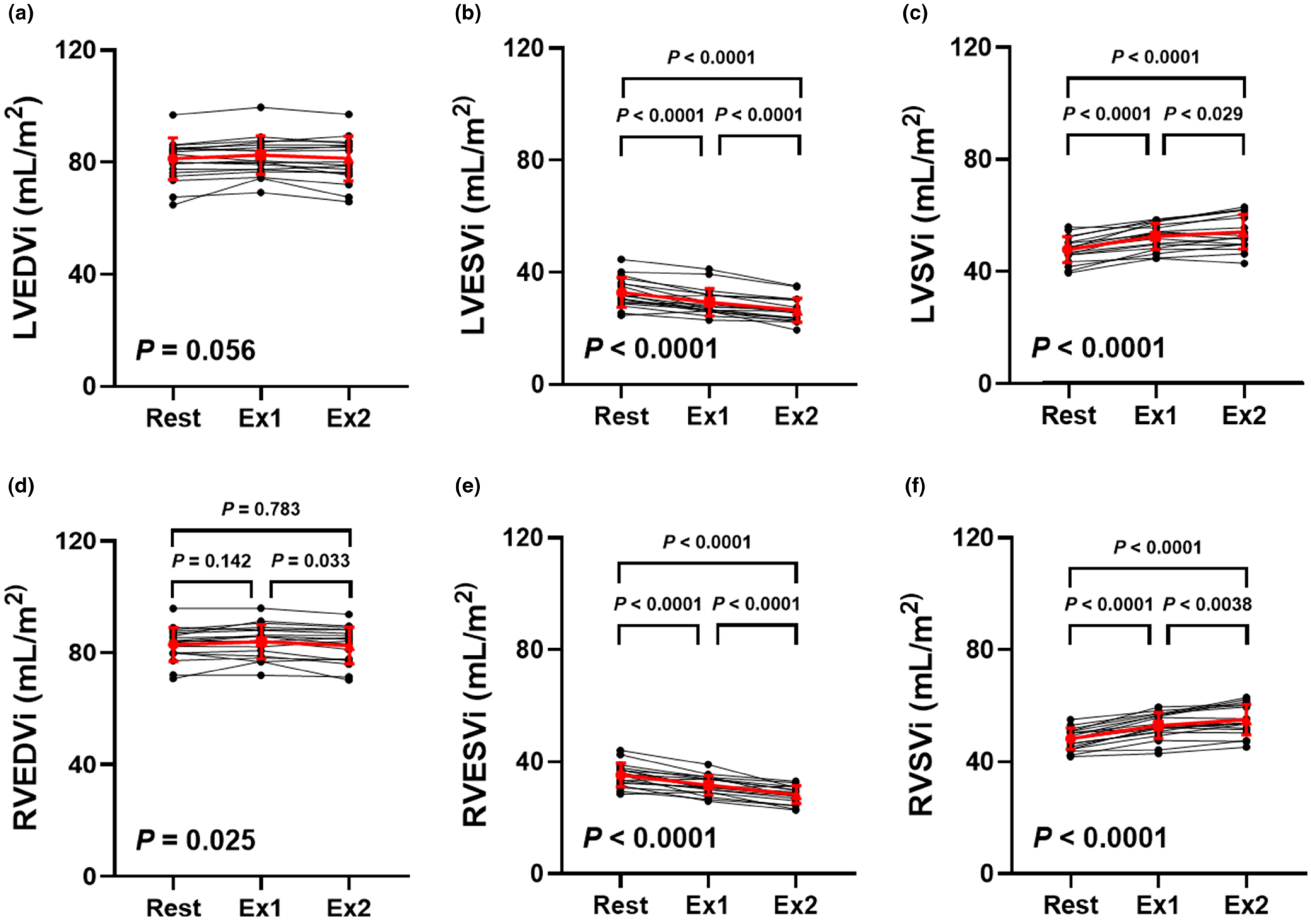
Comparison of changes in left and right ventricular volumes at rest and during exercise. (a) LVEDVi, (b) LVESVi, (c) LVSVi, (d) RVEDVi, (e) RVESVi, and (f) RVSVi. The black line represents the data for individual participants. The red line represents the mean. Data are presented as the mean ± SD and analyzed using repeated‐measures one‐way analysis of variance with Tukey's post hoc test. Statistical significance is set at *p* < 0.05. EDVi, end‐diastolic volume index; ESVi, end‐systolic volume index; Ex1, exercise at a target heart rate of 100 beats/min; Ex2, exercise at 40% of peak work rate; LV, left ventricle; RV, right ventricle; SVi, stroke volume index.

**TABLE 2:** Cardiovascular magnetic resonance imaging data on exercise.

	Rest	Ex1	Ex2	*p* value
Work rate (W)		33 ± 27	67 ± 22	
sBP (mmHg)	107 ± 14	124 ± 20	135 ± 18	<0.0001
dBP (mmHg)	60 ± 10	71 ± 17	77 ± 16	0.0077
HR (bpm)	66 ± 11	101 ± 10	113 ± 14	<0.0001
SpO_2_ (%)	97 ± 1	97 ± 1	97 ± 1	0.528
LVEDV (mL)	133.5 ± 21.8	135.7 ± 21.8	133.6 ± 22.4	0.043
LVEDVi (mL/m^2^)	80.5 ± 7.5	81.8 ± 7.0	80.7 ± 8.0	0.056
LVESV (mL)	54.5 ± 11.7	48.8 ± 11.0	44.0 ± 9.5	<0.0001
LVESVi (mL/m^2^)	32.8 ± 5.3	29.4 ± 4.9	26.5 ± 4.3	<0.0001
LVSV (mL)	79.1 ± 12.8	86.8 ± 13.6	89.6 ± 15.8	<0.0001
LVSVi (mL/m^2^)	47.7 ± 4.6	52.5 ± 4.7	54.1 ± 6.1	<0.0001
LVEF (%)	59 ± 4	64 ± 4	67 ± 4	<0.0001
RVEDV (mL)	137.6 ± 19.9	139.3 ± 20.0	137.2 ± 20.8	0.017
RVEDVi (mL/m^2^)	83.1 ± 6.0	84.2 ± 6.1	82.8 ± 6.6	0.025
RVESV (mL)	58.2 ± 10.4	52.2 ± 9.2	46.7 ± 7.9	<0.0001
RVESVi (mL/m^2^)	35.2 ± 4.2	31.5 ± 3.4	28.2 ± 3.2	<0.0001
RVSV (mL)	79.3 ± 11.3	87.1 ± 12.7	90.7 ± 14.7	<0.0001
RVSVi (mL/m^2^)	48.0 ± 3.8	52.7 ± 4.7	54.8 ± 5.4	<0.0001
RVEF (%)	58 ± 3	63 ± 3	66 ± 3	<0.0001
AoSV (mL)	72.4 ± 13.0	79.5 ± 13.8	82.0 ± 16.0	<0.0001
AoSVi (mL/m^2^)	43.7 ± 5.0	48.0 ± 4.7	49.4 ± 5.9	<0.0001
PASV (mL)	76.0 ± 16.0	86.3 ± 13.2	87.2 ± 15.5	<0.0001
PASVi (mL/m^2^)	46.7 ± 5.0	51.4 ± 4.4	52.6 ± 5.5	<0.0001
RV/LV EDV ratio	1.03 ± 0.04	1.03 ± 0.04	1.03 ± 0.04	0.677
RV/LV ESV ratio	1.09 ± 0.14	1.09 ± 0.12	1.08 ± 0.13	0.842

*Note*: Values are presented as the mean ± SD. Blood pressure data were available for 18 participants at rest and 17 participants at Ex1 and Ex2, with exercise measurements missing for one participant. *p* values represent comparisons across the three stages using a mixed‐effects model for blood pressure variables and repeated‐measures one‐way analysis of variance for all other variables.

Abbreviations: AoSV, aortic stroke volume; AoSVi, aortic stroke volume index; dBP, diastolic blood pressure; Ex1, exercise at a target heart rate of 100 beats/min; Ex2, exercise at 40% of peak work rate; HR, heart rate; LVEDV, left ventricular end‐diastolic volume; LVEDVi, left ventricular end‐diastolic volume index; LVEF, left ventricular ejection fraction; LVESV, left ventricular end‐systolic volume; LVESVi, left ventricular end‐systolic volume index; LVSV, left ventricular stroke volume; LVSVi, left ventricular stroke volume index; PASV, pulmonary artery stroke volume; PASVi, pulmonary artery stroke volume index; RV/LV EDV ratio, right‐to‐left ventricular end‐diastolic volume ratio; RV/LV ESV ratio, right‐to‐left ventricular end‐systolic volume ratio; RVEDV, right ventricular end‐diastolic volume; RVEDVi, right ventricular end‐diastolic volume index; RVEF, right ventricular ejection fraction; RVESV, right ventricular end‐systolic volume; RVESVi, right ventricular end‐systolic volume index; RVSV, right ventricular stroke volume; RVSVi, right ventricular stroke volume index; sBP, systolic blood pressure; SpO_2_, peripheral oxygen saturation.

RV/LV EDV ratio remained stable at 1.03 at rest, Ex1, and Ex2; RV/LV ESV ratio also remained stable at 1.09, 1.09, and 1.08, respectively. There were no significant differences in either ratio (Figure 3). Mean paired changes in RV/LV EDV and ESV ratios across exercise stages were small, with corresponding 95% confidence intervals and Cohen's *d*_z_ effect sizes provided in Table S3.

**FIGURE 3:**
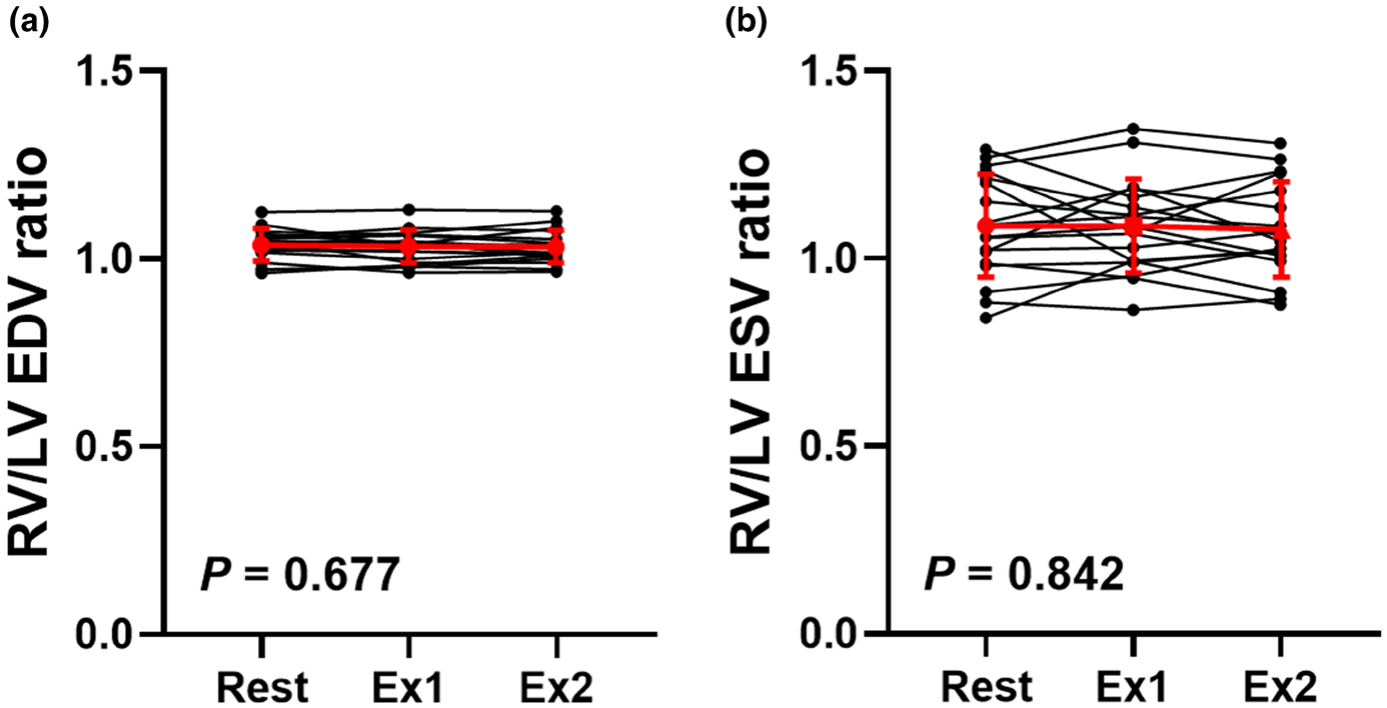
RV/LV EDV and ESV ratios at rest and during exercise stress. (a) Right ventricular end‐diastolic volume/left ventricular end‐diastolic volume, and (b) right ventricular end‐systolic volume/left ventricular end‐systolic volume. The black line represents the data for individual participants. The red line represents the mean. Data are presented as the mean ± SD and analyzed using repeated‐measures one‐way analysis of variance with Tukey's post hoc test. Statistical significance is set at *p* < 0.05. Ex1, exercise at a target heart rate of 100 beats/min; Ex2, exercise at 40% of peak work rate; RV/LV EDV ratio, right‐to‐left ventricular end‐diastolic volume ratio; RV/LV ESV ratio, right‐to‐left ventricular end‐systolic volume ratio.

## 4 DISCUSSION

This study demonstrated three principal findings. First, the RV/LV volume ratios at both end‐diastole and end‐systole remained stable from rest through exercise, supporting a preserved RV‐LV volumetric relationship in healthy adults during the exercise stages examined. Second, during low‐to‐moderate intensity exercise, stroke volume was increased in both ventricles predominantly through reduction in ESV rather than augmentation of EDV. Third, ECG‐gated exCMR enabled simultaneous assessment of RV and LV volumes during low‐to‐moderate supine exercise in healthy adults, while maintaining diagnostic image quality across all participants and internal concordance between volumetric and PC‐derived stroke‐volume estimates.

In previous reports, assessments of ventricular function during exercise have predominantly relied on echocardiography. Studies using exercise‐stress echocardiography have largely focused on LV function (Lancellotti et al., 2016), and relatively few have evaluated RV performance. Attempts to assess RV function have relied on surrogate indices, such as tricuspid annular plane systolic excursion, s', and RV fractional area change, rather than direct volumetric measurements (Chia et al., 2015; D'Alto et al., 2017; Pergola et al., 2021). Among exCMR studies, a study by Scharhag et al. compared biventricular hypertrophy in athletes but did not examine dynamic exercise responses (Scharhag et al., 2002). Claessen et al. investigated the influence of respiration on right and left ventricular volumes at rest and during exercise (Claessen et al., 2014). La Gerche et al. characterized biventricular volumetric responses across graded and high‐intensity exercise using real‐time CMR, with demonstrated reproducibility and validation against the direct Fick method (la Gerche et al., 2013). In light of these prior studies, the present study provides an ECG‐gated quantitative description of relative RV‐LV volumetric adaptation at end diastole and end systole during standardized submaximal supine exercise.

The present study used ECG‐gated cine CMR during exercise to quantify biventricular volumes at end diastole and end systole. This approach provides complementary methodological features alongside previously established real‐time exercise CMR approaches. First, CMR enables direct volumetric assessment of the morphologically complex RV without geometric assumptions. Second, the ECG‐gated approach used in the present study allowed consistent identification of end diastole and end systole during standardized submaximal exercise and provides complementary data to previously established real‐time exercise CMR approaches. Third, cine‐derived stroke volumes showed internal concordance with phase‐contrast flow‐derived measurements in the ascending aorta and main pulmonary artery, providing a non‐invasive internal consistency check within the present protocol. Given the expected matching of right‐ and left‐sided stroke volumes in a series circulation, the present study was not intended to re‐establish this physiological principle. Rather, its contribution is the quantitative characterization of RV/LV EDV and ESV ratios across standardized submaximal exercise stages in healthy adults, providing physiological reference data for future disease‐focused studies.

A constant RV/LV volume ratio during exercise reflects the coordinated adaptation of both vascular afterload and ventricular performance. RV and LV volume responses during exercise are influenced by pulmonary and systemic vascular loading. Accordingly, a stable RV/LV volume ratio may, in part, reflect proportional changes in pulmonary vascular resistance (PVR) and systemic vascular resistance (SVR). However, ventricular volume responses cannot be attributed solely to vascular loading, as they reflect the integrated effects of preload, afterload, contractility, heart rate, and pericardial constraint. Within this integrated physiological framework, exercise‐related changes in pulmonary and systemic vascular loading likely contribute to RV and LV chamber adaptation in healthy adults. In this setting, stroke‐volume augmentation in both ventricles occurred predominantly through reductions in ESV rather than substantial increases in EDV. This response pattern suggests that enhanced contractility, together with vascular adaptation, played an important role in supporting the increased cardiac output observed during exercise.

At the same time, an increase in stressed blood volume elevates venous return, shifting both the venous return curve and the Frank–Starling relationship upward/rightward. Thus, the operating point moves to the right and upward along an improved cardiac function curve. Schweitzer et al. reported that stroke‐volume augmentation following supine exercise was predominantly mediated by a reduction in ESV, with a smaller increase in EDV (Schweitzer et al., 2025). This response pattern is broadly consistent with the present study, although EDV changed little over the workload range examined here. The modest EDV increase in their study may partly reflect the higher exercise intensity compared with our low‐to‐moderate intensity protocol, although differences in image acquisition timing—immediately after exercise cessation versus during ongoing exercise—also limit direct comparison. The limited EDV response observed in the present study may also reflect the supine posture, which increases central preload at rest and may limit further preload augmentation during exercise. This posture‐dependent preload condition differs from upright exercise and should be considered when interpreting exercise‐related ventricular volume responses. Recent proof‐of‐concept work demonstrated that lower‐body negative pressure during supine exCMR restores larger exercise‐related EDV and stroke‐volume responses by reintroducing orthostatic stress (Hathorn et al., 2025).

The preservation of the RV/LV ratio under these conditions suggests that the septal geometry and position remain relatively constant, allowing both ventricles to benefit equally from exercise‐induced inotropic enhancement. This balanced biventricular response represents coordinated cardiovascular efficiency in a healthy state. However, the present study did not directly assess septal geometry, respiratory phase, ventricular pressures, or pericardial constraint. Therefore, these findings should not be interpreted as direct mechanistic proof of systolic or diastolic ventricular interaction. Nonetheless, alterations in ventricular interdependence may become clinically relevant under pathological conditions characterized by RV volume or pressure overload. RV enlargement and altered septal mechanics have been described in pulmonary hypertension and may disrupt diastolic ventricular interaction (Olsen et al., 1983; Vonk Noordegraaf et al., 2017). In contrast, the present study demonstrated a preserved relative RV‐LV volumetric relationship across the exercise stages examined in healthy adults. These findings should primarily be interpreted as physiological reference data for healthy adults studied during low‐to‐moderate supine exercise. They may inform future investigations in pulmonary vascular disease or heart failure with preserved ejection fraction, in which exercise‐related alterations in RV‐LV adaptation are biologically plausible. However, disease‐specific relevance remains to be established through direct patient studies.

This study had some limitations that warrant consideration. First, our sample size (*n* = 18) was modest, although there was adequate statistical power to detect a lack of change in the RV/LV ratios (beta error <0.20). Second, the exercise stages were limited to the submaximal low‐to‐moderate range, including a target HR of 100 bpm and 40% of peak work rate, and were lower than the intensities achieved in some previous real‐time exercise CMR studies (la Gerche et al., 2013; Le et al., 2017; Schweitzer et al., 2025). The present protocol was designed to characterize biventricular volumetric responses under standardized, clinically applicable submaximal supine exercise conditions rather than to assess cardiovascular responses at high or near‐maximal exercise intensities. Such submaximal exercise conditions may also be relevant to future studies of patient populations in whom high‐intensity exercise is not feasible. Accordingly, whether the stable RV‐LV volumetric relationship observed here persists at higher exercise intensities remains to be established. Third, the absence of invasive hemodynamic measurements precludes the direct correlation of volume ratios with PVR/SVR ratios. However, the non‐invasive nature of exCMR enhances its potential for repeated assessments and broader clinical applicability. Fourth, although exercise CMR feature‐tracking has been shown to be feasible (Lin et al., 2019), current feature‐tracking methods may be less robust during free‐breathing exercise because of substantial respiratory motion and elevated heart rates (Morales et al., 2026). Accordingly, myocardial deformation parameters, including strain and twist/torsion, were not evaluated because the present protocol prioritized continuous exercise and free‐breathing image acquisition to preserve physiological exercise conditions. Finally, we studied a relatively young cohort of healthy adults, and age‐related changes in cardiac structure and cardiovascular reserve may influence biventricular volumetric responses to exercise. Accordingly, the applicability of these findings to older individuals remains to be established in dedicated studies.

In healthy adults, ECG‐gated exCMR demonstrated stable RV/LV EDV and ESV ratios during low‐to‐moderate intensity supine exercise, with stroke volume augmentation occurring predominantly through reduced ESV. These findings support a preserved relative RV‐LV volumetric relationship across the exercise stages examined in the present protocol and provide quantitative physiological reference data for future disease‐focused studies.

## AUTHOR CONTRIBUTIONS

**Megumi Ikeda:** Conceptualization; data curation; formal analysis; investigation; methodology; validation; visualization. **Michinari Hieda:** Conceptualization; data curation; validation; visualization. **Keimei Yoshida:** Conceptualization; data curation; funding acquisition; investigation; project administration; validation. **Misaki Imabayashi:** Conceptualization; data curation; investigation. **Daisuke Toyomura:** Conceptualization; investigation; methodology. **Ryo Izumi:** Conceptualization; data curation; validation. **Akiko Nishizaki:** Conceptualization; validation. **Tatsuhiro Wada:** Conceptualization; data curation; investigation. **Yuya Saito:** Conceptualization; data curation; investigation. **Chiaki Tokunaga:** Conceptualization; data curation; investigation. **Kei Nishikawa:** Conceptualization; data curation; investigation. **Saki Kurokawa:** Conceptualization; data curation; investigation. **Yuya Fukudome:** Conceptualization; data curation; investigation. **Takuya Hino:** Investigation. **Yuzo Yamasaki:** Conceptualization; funding acquisition; investigation; methodology. **Kazuya Hosokawa:** Funding acquisition. **Mitsuhiro Fukata:** Project administration. **Kousei Ishigami:** Funding acquisition; project administration; supervision. **Kohtaro Abe:** Conceptualization; funding acquisition; project administration. **Koichi Akashi:** Project administration.

## FUNDING INFORMATION

This work was supported by research grants from Konica Minolta, Inc., Japanese Society of Grant for Doctors for Clinical Research, and Suzuken Memorial Foundation.

## CONFLICT OF INTEREST STATEMENT

Y.Y., K.H., K.I., and K.A. received research grants from Konica Minolta Inc. K.Y. received research grants from Japanese Society of Grant for Doctors for Clinical Research and Suzuken Memorial Foundation. The remaining authors declare no conflicts of interest.

## Supporting information


**Table S1:** Sex‐stratified participant characteristics.
**Table S2:** Sex‐stratified stage‐specific biventricular volumetric measurements indexed to body surface area.
**Table S3:** RV/LV ratio changes with effect sizes and 95% CI.

## Data Availability

The source data will be made available by the corresponding author upon reasonable request and justification.
