# Supplementary material for: RV‐LV volumetric relationship during graded supine exercise in healthy adults assessed by cardiovascular magnetic resonance
Source: Physiol Rep. 2026 Nov 6;14(21):e71206. doi: 10.14814/phy2.71206 (PMC13740968; doi:10.14814/phy2.71206)
Supplement: Supplementary file 1 [file PHY2-14-e71206-s001.docx]

**Supplementary Table S1.** **Sex-stratified participant characteristics**

| **Baseline characteristics** | | |
| --- | --- | --- |
|  | Male (n=9) | Female (n=9) |
| Age | 37 ± 11 | 34 ± 11 |
| Height (cm) | 170.6 ± 5.6 | 159.4 ± 6.5 |
| Weight (kg) | 67.8 ± 8.9 | 51.8 ± 5.0 |
| BSA (m²) | 1.79 ± 0.13 | 1.52 ± 0.08 |
| **CPX** | | |
| RER | 1.29 ± 0.06 | 1.30 ± 0.11 |
| Peak work rate (W) | 195 ± 38 | 128 ± 32 |
| Peak VO₂ (mL/kg/min) | 34.4 ± 4.7 | 26.4 ± 5.9 |
| Pred peak VO_2_ (%) | 107.0 ± 13.6 | 92.4 ± 18.3 |

Values are presented as the mean ± SD.
Abbreviations: BSA, body surface area; RER, respiratory exchange ratio; Peak VO2, peak oxygen uptake; Pred peak VO2, predicted peak oxygen uptake; VE/VCO2 slope, ventilatory equivalent for carbon dioxide slope; CPX, cardiopulmonary exercise testing.

**Supplementary Table S2. Sex-stratified stage-specific biventricular volumetric measurements indexed to body surface area**

|  | **Male** |  |  | **Female** |  |  |
| --- | --- | --- | --- | --- | --- | --- |
|  | **Rest** | **Ex1** | **Ex2** | **Rest** | **Ex1** | **Ex2** |
| LVEDVi (mL/m^2^) | 82.8 ± 8.1 | 84.5 ± 8.6 | 83.6 ± 9.0 | 78.3 ± 6.3 | 79.2 ± 3.6 | 77.6 ± 5.7 |
| LVESVi (mL/m^2^) | 35.0 ± 5.6 | 31.4 ± 5.7 | 28.2 ± 4.7 | 30.7 ± 4.2 | 27.4 ± 2.8 | 24.8 ± 3.2 |
| LVSVi (mL/m^2^) | 47.9 ± 4.7 | 53.1 ± 5.7 | 55.3 ± 7.2 | 47.6 ± 4.9 | 51.8 ± 3.7 | 52.8 ± 5.0 |
| LVEF (%) | 58 ± 4 | 63 ± 4 | 66 ± 4 | 61 ± 4 | 65 ± 3 | 68 ± 3 |
| RVEDVi (mL/m^2^) | 84.7 ± 6.8 | 86.2 ± 7.3 | 85.2 ± 6.8 | 81.6 ± 5.1 | 82.2 ± 4.2 | 80.4 ± 5.7 |
| RVESVi (mL/m^2^) | 36.7 ± 4.4 | 33.5 ± 3.0 | 29.5 ± 2.1 | 33.7 ± 3.5 | 29.5 ± 2.6 | 26.9 ± 3.6 |
| RVSVi (mL/m^2^) | 48.0 ± 4.2 | 52.8 ± 6.1 | 55.7 ± 6.3 | 47.9 ± 3.5 | 52.7 ± 3.4 | 53.8 ± 4.5 |
| RVEF (%) | 58 ± 3 | 61 ± 3 | 65 ± 3 | 59 ± 3 | 64 ± 2 | 67 ± 3 |
| RV/LV EDV ratio | 1.02 ± 0.03 | 1.02 ± 0.04 | 1.02 ± 0.05 | 1.04 ± 0.05 | 1.04 ± 0.05 | 1.04 ± 0.04 |
| RV/LV ESV ratio | 1.06 ± 0.14 | 1.09 ± 0.14 | 1.06 ± 0.13 | 1.11 ± 0.14 | 1.08 ± 0.11 | 1.09 ± 0.12 |

Values are presented as the mean ± SD.

Abbreviations: LVEDVi, left ventricular end-diastolic volume index; LVESVi, left ventricular end-systolic volume index; LVSVi, left ventricular stroke volume index; LVEF, left ventricular ejection fraction; RVEDVi, right ventricular end-diastolic volume index; RVESVi, right ventricular end-systolic volume index; RVSVi, right ventricular stroke volume index; RVEF, right ventricular ejection fraction; RV/LV EDV ratio, right-to-left ventricular end-diastolic volume ratio; RV/LV ESV ratio, right-to-left ventricular end-systolic volume ratio; Ex1, exercise at a target heart rate of 100 beats/min; Ex2, exercise at 40% of peak work rate.

**Supplementary Table S3. RV/LV ratio changes with effect sizes and 95% CI**

|  | **Comparison** | **Mean paired difference** | **95% CI** | **Cohen’s d_z_** |
| --- | --- | --- | --- | --- |
| **RV/LV EDV ratio** | **Ex1 − Rest** | -0.0043 | -0.017 to 0.008 | -0.165 |
|  | **Ex2 − Rest** | -0.005 | -0.019 to 0.010 | -0.162 |
|  | **Ex2 − Ex1** | -0.0004 | -0.012 to 0.011 | -0.018 |
| **RV/LV ESV ratio** | **Ex1 − Rest** | -0.0002 | -0.050 to 0.049 | -0.002 |
|  | **Ex2 − Rest** | -0.010 | -0.062 to 0.041 | -0.100 |
|  | **Ex2 − Ex1** | -0.010 | -0.048 to 0.027 | -0.134 |

Values are presented as mean paired differences, 95% confidence intervals (95% CI), and Cohen’s d_z_ effect sizes.
Abbreviations: RV/LV EDV ratio, right-to-left ventricular end-diastolic volume ratio; RV/LV ESV ratio, right-to-left ventricular end-systolic volume ratio; Ex1, exercise at a target heart rate of 100 beats/min; Ex2, exercise at 40% of peak work rate.
